# Dr. Murthy J. R. Kolluri

**DOI:** 10.4103/0970-2113.80347

**Published:** 2011


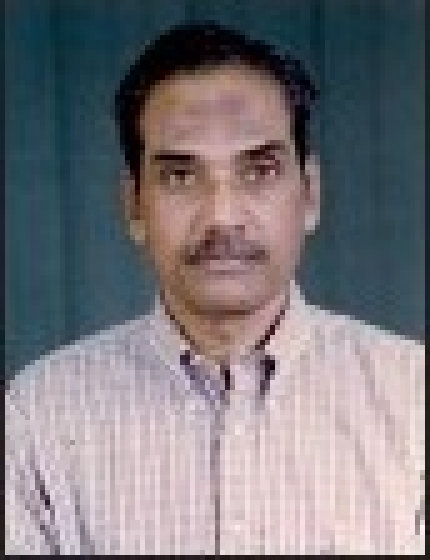


Dr. Murthy J. R. Kolluri

(From 19^th^October, 1939 to 25^th^February, 2011)

The eminent chest physician, Dr. K. J. R. Murthy, was a doyen of respiratory medicine and a pillar of the Indian Chest Society. He has made a huge contribution in the field of tuberculosis.

He obtained his MBBS from Gandhi Medical College, Hyderabad, in 1961 and did his Post Graduation in Internal Medicine from Guntur Medical College, Guntur, in 1967. After acquiring the postgraduate degree, he immediately joined as Assistant Professor of Medicine at Osmania Medical College, Hyderabad. After serving there for 12 years, he moved to Bhagwan Mahaveer Hospital and Research Centre, Hyderabad, as a Consultant Chest Physician and subsequently became its Medical Director in 2005. He worked at the same institution as Director, Mahaveer PPM DOTS, till his last days.

He distinguished himself as a popular teacher, good administrator and an excellent clinician. He was associated with several operational research studies for the control of tuberculosis. Dr. Murthy was member of A.P.I., A.C.C.P., and TB Association of India. He was in the Editorial Board of *“Lung India”* and was one of the founding members of Indian Chest Society.

He was recipient of several awards including Mahaveer Mahatma Award 2005, and conducted research projects funded by WHO, Overseas Development Agency UK, University of Newcastle UK, and Government of India. He was involved in many community welfare activities.

Dr. Murthy has to his credit 126 publications in national and international journals, 115 presentations and many a contributions to books and updates. He was invited as a guest speaker to almost all countries of the world and acted as Panelist and Session Chairman in many world congresses.

In view of his tremendous contributions to various organizations and to Indian Chest Society, he was highly respected as an elder statesman and his invaluable advice was sought on various occasions. His knowledge regarding constitutional intricacies was exemplary. May God Almighty give eternal peace to the departed soul!

We are grateful to Prof. S. R. Kamat, Former Head of Respiratory Medicine at the King Edward Memorial Hospital, Mumbai, for providing material and inspiring us to get this document prepared. The obituary was prepared with courtesy of Prof. U. S. Mathur, Former Head of Division of Allergy and Pulmonary Medicine, SMS Medical College, Jaipur, and Prof. Sundaram Challa, Head of Department of Pathology, Nizams’ Institute of Medical Sciences, Hyderabad. *Lung India* Editorial Board deeply regrets this irremediable loss and expresses sincere regards to grieving family, relatives and colleagues.

